# AdductHunter: identifying protein-metal complex adducts in mass spectra

**DOI:** 10.1186/s13321-023-00797-7

**Published:** 2024-02-06

**Authors:** Derek Long, Liam Eade, Matthew P. Sullivan, Katharina Dost, Samuel M. Meier-Menches, David C. Goldstone, Christian G. Hartinger, Jörg S. Wicker, Katerina Taškova

**Affiliations:** 1 School of Computer Science, University of Auckland, 1010 Auckland, New Zealand; 2 School of Chemical Sciences, University of Auckland, 1142 Auckland, New Zealand; 3 School of Biological Sciences, University of Auckland, 1142 Auckland, New Zealand; 4 Department of Engineering Science, University of Auckland, 1010 Auckland, New Zealand; 5 Department of Analytical Chemistry, Faculty of Chemistry, University of Vienna, 1090 Vienna, Austria

**Keywords:** Mass spectrometry, Protein adducts, Constraint integer optimization, Dynamic time warping

## Abstract

Mass spectrometry (MS) is an analytical technique for molecule identification that can be used for investigating protein-metal complex interactions. Once the MS data is collected, the mass spectra are usually interpreted manually to identify the adducts formed as a result of the interactions between proteins and metal-based species. However, with increasing resolution, dataset size, and species complexity, the time required to identify adducts and the error-prone nature of manual assignment have become limiting factors in MS analysis. AdductHunter is a open-source web-based analysis tool that  automates the peak identification process using constraint integer optimization to find feasible combinations of protein and fragments, and dynamic time warping to calculate the dissimilarity between the theoretical isotope pattern of a species and its experimental isotope peak distribution. Empirical evaluation on a collection of 22 unique MS datasetsshows fast and accurate identification of protein-metal complex adducts in deconvoluted mass spectra.

**Supplementary Information:**

The online version contains supplementary material available at 10.1186/s13321-023-00797-7.

## Introduction

Mass spectrometry (MS) is a well-established analytical technique for chemical identification and molecular weight determination of various analytes [1]. The experimental output is a mass spectrum consisting of intensity values at corresponding mass-to-charge ratios (m/z). Analysis of small molecules by electrospray ionization (ESI)-MS, one of the most widely used MS techniques, results in mostly singly-charged ions. In the case of proteins or other biomolecules, which have much higher molecular weights, charge state envelopes are formed from ions at different charge states, but originate from the same molecule. The isotopes of the elements present in the protein and its adducts change the isotope peak pattern for each peak, forming Gaussian-type profiles. Due to the complexity of such spectra, maximum entropy deconvolution [2, 3] as a pre-processing step facilitates the analysis of proteins reconstituting the charge state envelope for each species detected into neutral mass peaks.

MS has proven particularly valuable in characterizing metallodrug interactions with proteins, e.g., protein-metal complex stoichiometry, adduct composition, binding sites, and structural changes [4–12]. For current metallodrugs to progress toward clinical development, it is crucial to understand the pharmacological properties, notably metallodrug-protein interactions [13–15].

However, interpreting mass spectra is typically done manually, which can be time-consuming, tedious, and error-prone due to the complexity of mass spectra, in particular for reactive species that can undergo changes not only upon interacting with proteins, but also by reaction with matrix components or during the analysis process with solvent molecules.

Software solutions have been explored to automatize the identification of protein adducts, but for example, Analysis of Protein Modifications from Mass Spectra ($\hbox {Apm}^2$s) [16] is targeted at proteomics workflows, pyOpenMS [17] is a mass spectrometry-based proteomics analysis tool but not specifically designed for identifying protein-metal complex adducts. The Nesvizhskii lab and collaborators have created a suite of software^1^ for proteomics and metabolomics applications [18–22] which are well supported for these applications. mMass [23] and pyQms [24] are either again focused on proteomics or metabolomics, and limited to a narrow set of inputs, or have had no further development and support in recent years.

Therefore, AdductHunter is introduced here as a web-based tool that automates the identification of protein-metal complex adducts in deconvoluted mass spectra, which, to the best of our knowledge, is the first tool of its kind for this purpose.

## Implementation

**Fig. 1:**
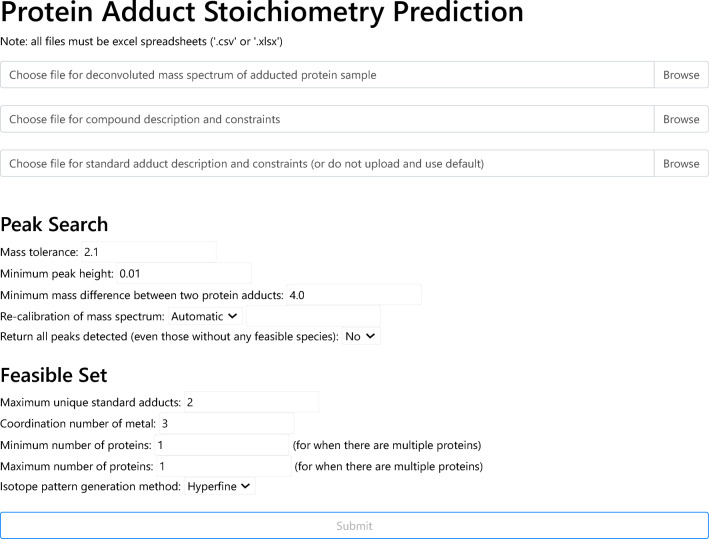
Webpage layout showing input files and parameters for peak identification and the constraint optimization formulation

**Fig. 2:**
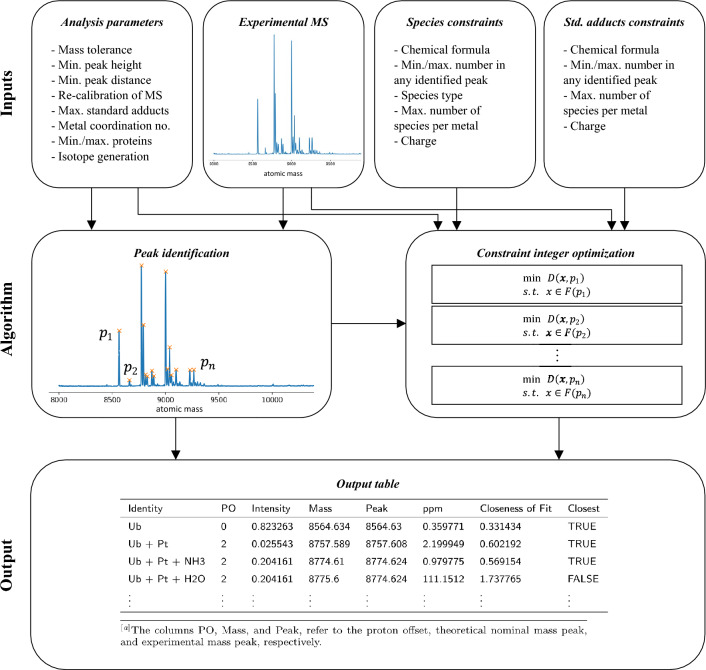
Overview of the underlying algorithm to AdductHunter

AdductHunter is a web-based tool that automates the identification of protein adducts in deconvoluted mass spectra (see Fig. 1). It requires a series of input files and parameters, returning a downloadable output file that contains a list of (feasible) species corresponding to different peaks in the input spectrum (see Fig. 2 for its general algorithm). These species are sorted by their similarity to the experimental peaks as scored by closeness of fit (loss) to isotope pattern and mass error.

AdductHunter is freely accessible on GitHub^2^ under an MIT license or at adducthunter.wickerlab.org and was created using Python 3. Hence, it is dependent on several Python packages, namely pyOpenMS [17], ORTools [25], SciPy [26], and Flask [27]. In this section, we outline the specifics behind AdductHunter’s implementation, alongside examples of a well-studied protein/metallodrug system [4, 5, 28–30], namely ubiquitin (Ub) incubated with cisplatin (cis-Pt($\hbox {NH}_3$)_2_$\hbox {Cl}_2$), to provide clarity on usage.

### Input files

Three input files are required; (1) the deconvoluted mass spectrum, for example obtained using Maximum Entropy Deconvolution in Bruker DataAnalysis to produce a charge neutral spectrum [2, 3]; (2) a file that lists the protein and any atoms, ions, and solvents contained in the sample and their corresponding constraints, such as charge and coordination number, the number of expected adducts formed; and (3) a description of the standard adducts involved in the sample and their corresponding constraints, which is expected to be very similar for most experiments. These are required to be of .xlsx or .csv file types and assumed to have the correct formatting (see Tables 1, 2, 3 and Additional Files 1 and 2 for examples of the expected layout for these input files).

**Table 1:** Input file for a deconvoluted mass spectrum in the mass range of 8000–11,000 recorded for a mixture of Ub and cisplatin containing mass, *m*, and intensity, *I*, values

#	*m*(*Da*)	*I*
1	8000	419733
2	8000.561	215877
3	8001.474	168996
$\vdots$	$\vdots$	$\vdots$
2225	10997.12	116811
2226	10998.31	231463
2227	10999.56	231444

Intensities can be of any unit as they will be normalized relative to the most abundant peak

**Table 2:** Species description and constraints input table for a sample containing the protein ubiquitin (Ub) and cisplatin

Species	Formula	Min	Max	Type	M	Charge
Ubiquitin	C378H629N105O118S1	1	1	Protein		0
Platinum	Pt	0	3	Metal		2
Ammonia	NH3	0	6	Other	2	0
Water	H2O	0	3	Other	2	0
Chlorine	Cl	0	6	Other	2	− 1

Min, Max indicate the minimal and maximal values for each component in any identified peak

M refers to the maximum number of the corresponding coordinating species per metal

An empty value means there is no limit, which is expected here as these adducts do not coordinate to the metal centre

The charge needs to be provided as positive or negative integers

**Table 3:** Input table of standard adducts and constraints for the ubiquitin and cisplatin system

Species	Formula	Min	Max	M	Charge
Hydrogen	H	0	10		1
Sodium	Na	0	1		1
Lithium	Li	0	1		1
Potassium	K	0	1		1

Min and Max indicate each component’s minimal and maximal values in any identified peak

M refers to the maximum number of the corresponding coordinating species per metal

An empty value means there is no limit, which is expected here as these adducts do not coordinate to the metal centre

The charge needs to be provided as positive or negative integers

### Peak identification

AdductHunter begins by identifying peaks within the mass spectrum. Users are required to specify three parameters involved in this process: (1) the (normalized) noise threshold or minimum peak height; (2) the minimum distance between adjacent peaks in atomic mass units; and (3) whether a linear re-calibration of the spectrum is required using known peaks as internal standards. In the case that a re-calibration is applied, all mass-to-charge values are shifted equally, either according to the difference between the (theoretical) peak isotopic mass of the protein and the closest identified isotopologue peak in the mass spectrum, or a user-specified value.

**Fig. 3:**
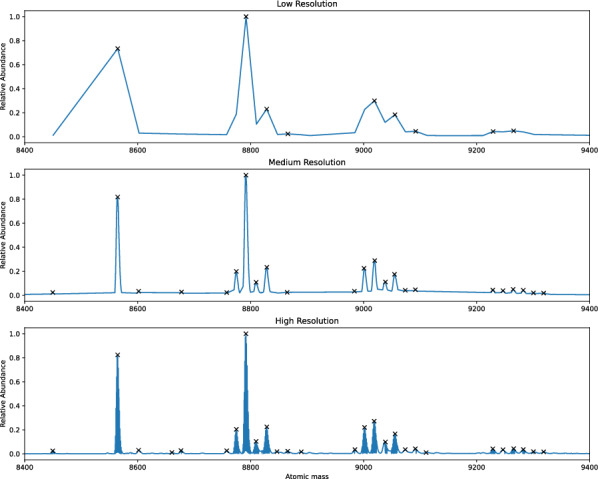
Peaks identified in mass spectra recorded for ubiquitin (Ub) and cisplatin mixtures at low, medium, and high resolution

With these parameters set, peaks are identified in a two-step process. The spectrum intensities are first normalized to the most abundant peak, then peaks exceeding the minimum height threshold are identified using SciPy’s peak detection function, which yielded similar results to several recently reported MS peak detection algorithms [31–33]. Higher-resolution MS, however, picks up a much greater number of low intensity peaks, leading to more peaks having an intensity larger than the noise threshold and a significant number of false positive peaks. As a result, a second filtering step was included in the peak identification process. Filtering uses the minimum distance between peaks to remove peaks belonging to the same species, ensuring isotope peaks within the same isotope pattern are only detected once, a feature increasingly relevant in mass spectra collected with higher resolution instruments (see Fig. 3). Additionally, users can specify to only return detected peaks with at least one feasible species in the output.

The peak isotopic mass refers to the highest nominal mass peak by intensity-weighted average of the hyperfine mass distribution (at each integer mass) of a species. The most abundant isotopologue typically matches that of commercial isotope pattern predictors on the scale of $10^{-3}$ parts per million (ppm), e.g., the Bruker isotope pattern generator [34]. These mass values are later used in the constraint optimization formulation to linearly approximate the true mass value of a species.

### Optimization problem

Once peaks within the mass spectrum have been detected, AdductHunter will determine their corresponding speciations by formulating an optimization problem, involving an objective function subject to a set of constraints, at each identified peak *p*. The objective function measures the dissimilarity (distance) between the theoretical isotope pattern of a given species and the experimental isotope distribution of the peak. The constraints are established from user-defined parameters and files, forming the set of feasible solutions. In the context of this problem, a feasible solution refers to a combination of input compounds that gives a potential species matching the peak-centred experimental isotopic distribution. This gives the general formulation:

1 $$\begin{aligned} \begin{array}{ll} \text {min} &{} \displaystyle \phi (\varvec{x}, p) \\ \text {s.t.} &{} \varvec{x} \in F(p) \end{array} \end{aligned}$$

where $\varvec{x}$ is the vector of the number of molecules for each compound, $\phi (\varvec{x},p)$ is the dissimilarity between species $\varvec{x}$ and the experimental distribution around peak *p*, and *F*(*p*) is the set of feasible species at peak *p*. For example, to represent UbPt$($ $\hbox {NH}_3)_2$, we would have $\varvec{x} = (x_{\text {Ub}}, x_{\text {Pt}}, x_{\text {NH}_3}, \cdots )^T = (1, 1, 2, 0, \cdots , 0)^T$.

Due to noise and inaccuracies in the collection and averaging of mass spectra, the true species may not be optimal, that is, there exists another species that has an isotope pattern more similar to the experimental isotope distribution. However, the correct species is highly likely to be contained within the feasible set, if sensible constraints and parameters have been provided. Thus, returning all feasible species is helpful for post-optimization validation and analysis.

#### Constraint integer optimization formulation

A constraint integer optimization (CIO) formulation is a type of integer optimization formulation where all feasible integer solutions are returned. The formulation takes advantage of the problem structure and constraints to ensure sensible species are generated. Although once thought to be intractable, it has shown great advances in efficiency and speed in recent years, and can be solved quickly using industry-grade solvers such as CPLEX [35] and GUROBI [36], much faster than enumerating all possible solutions.

To start, the decision variables, $x_i$, are defined as the number of molecules present for protein/adduct *i*, each having a mass of $m_i$, for every *i* in the set of all species, *C*. The mass value here takes into account the charge discrepancy from adding a metal-based fragment, $c_i$, of species *i* by removing the mass of $c_i$ protons from its peak isotopic mass, that is,

2 $$\begin{aligned} m_i = P_i - 1.007825c_i, \quad \forall i \in C, \end{aligned}$$

where $P_i$ is the most abundant isotopologue of the species.

Constants/parameters in the formulation are defined in either the web application or the compound constraint files. The user-defined parameters in the web application are as follows:

i. The peak tolerance, *t*, defined as the neighbourhood of mass values around a peak *p*, at which a combination of species forms a feasible protein adduct. It is enforced by the constraint:
  3 $$\begin{aligned} p - t \le \displaystyle \sum \limits _{i \in C} m_{i}x_{i} \le p + t \end{aligned}$$
ii. The maximum number of unique standard adducts, *r*, in any feasible solution. We define standard adducts as those adducts frequently observed in ESI-MS, that is, the alkali metal ions Na+, Li+ and K+, as well as H+. To enforce this, the indicator variables $d_s=\mathbb {1}\{$standard adduct *s* is selected$\}$, to track which standard adducts have been selected, will need to be added with the following constraint:
  4 $$\begin{aligned} \displaystyle \sum \limits _{s \in S} d_{s} \le r, \end{aligned}$$
  where *S* is the set of all standard adducts and
  5 $$\begin{aligned} \mathbbm {1}\{X\}:= {\left\{ \begin{array}{ll} 1 &{} \text {if}\ X \text { is true,} \\ 0 &{} \text {otherwise.} \end{array}\right. } \end{aligned}$$
iii. The minimum, g, and maximum, h, number of proteins in a multi-protein use case, in any feasible solution. Again, the indicator variables $z_a=\mathbb {1}\{$primary *a* is selected$\}$, to track which primaries have been selected, will need to be added with the following constraint:
  6 $$\begin{aligned} g \le \displaystyle \sum \limits _{a \in A} z_{a} \le h, \end{aligned}$$
  where *A* is the set of all primaries.
iv. The coordination number, *v*, of metal *k* used. The coordination number for linear complexes is 2, for square planar and tetrahedral complexes is 4, and for octahedral complexes is 6, to name a few. For cisplatin, the platinum (II) metal center has $v=4$. AdductHunter supports one type of metal at a time. This constraint is enforced by:
  7 $$\begin{aligned} \displaystyle \sum \limits _{i \in C \setminus \{k, S\}} x_{i} \le vx_{k} \end{aligned}$$
  For the compound description/constraint files (see Tables 2, 3), the user-defined parameters are as follows:
v. The lower and upper bounds $l_i$ and $u_i$, respectively, for species *i* in any feasible solution, enforced by constraints:
  8 $$\begin{aligned} l_{i} \le x_{i} \le u_{i}, \quad \forall i \in C \end{aligned}$$
vi. The maximum number of coordinating species, $n_j$, per metal *k* for each binding species *j*, enforced by the constraint:
  9 $$\begin{aligned} x_{j} \le n_{j}x_{k}, \quad \forall j \in B, \end{aligned}$$
  where *B* is the set of all binding compounds.

Putting all of this together, along with non-negativity constraints, the final CIO formulation defining the feasible set *F*(*p*) at peak *p* is:

10 $$\begin{aligned} \begin{aligned} p - t \le \displaystyle \sum \limits _{i \in C} m_{i}x_{i} \le p + t \\ \displaystyle \sum \limits _{s \in S} d_{s} \le r \\ g \le \displaystyle \sum \limits _{a \in A} z_{a} \le h \\ \displaystyle \sum \limits _{i \in C \setminus \{k, S\}} x_{i} \le vx_{k} \\ l_{i} \le x_{i} \le u_{i}, \quad \forall i \in C \\ x_{j} \le n_{j}x_{k}, \quad \forall j \in B \\ d_s \in \{0, 1\} \quad \forall s \in S \\ z_a \in \{0, 1\} \quad \forall a \in A \\ x_{i} \in \mathbb {Z}_{\ge 0}, \quad \forall i \in C \end{aligned} \end{aligned}$$

As an example, we illustrate in the system involving ubiquitin incubated with cisplatin the CIO formulation defining the feasible set at the peak corresponding to a mass of 8774.6028 Da:

11 $$\begin{aligned} \begin{aligned} 8772.6028 \le m_{\text {Ub}}x_{\text {Ub}}+\cdots +m_{\text {K}}x_{\text {K}} \le 8776.6028 \\ d_{\text {Li}}+d_{\text {Na}}+d_{\text {K}} \le 2 \\ x_{\text {Ub}}+\cdots +x_{\text {Cl}} \le 4x_{\text {Pt}} \\ 1\le x_{\text {Ub}}\le 1,..., 0\le x_{\text {K}}\le 2 \\ x_{\text {NH}_3}, x_{\text {H}_2\text {O}}, x_{\text {Cl}} \le 2x_{\text {Pt}} \\ d_s \in \{0, 1\} \quad \forall s \in S \\ x_{\text {Ub}}, x_{\text {Pt}},..., x_{\text {K}} \in \mathbb {Z}_{\ge 0} \end{aligned} \end{aligned}$$

where the set of all species $C = \left\{ {{\text{Ubiquitin}},{\text{Platinum}},{\text{Ammonia,}} \ldots ,{\text{Potassium}}} \right\}$, the set of binding compounds $B = \left\{ {{\text{Ammonia}},{\text{Water}},{\text{Chlorine}}} \right\}$, the set of standard adducts $S = \left\{ {{\text{Lithium}},{\text{Sodium}},{\text{Potassium}}} \right\}$, the peak tolerance $t = 2$, the maximum number of unique standard adducts $r = 2$, the metal *k* is Platinum with a coordination number $v = 4$, and the maximum number of coordinating species is $n_j = 2$ for all binding compounds $j\in B$. Notice that since there is only one protein in this system, we do not have the multi-protein constraint.

#### Objective function

With the constraints established, we require an objective function that measures the similarity in shape and mass between theoretical and experimental isotope distributions, or the dissimilarity assuming a minimization problem. Furthermore, the effects of preceding and succeeding noisy peaks far from the peak for the most abundant isotopologue should be ignored, as well as intensities below a certain height due to the noise in high-resolution data—these do not help the measurement of similarity. Thus, only values within a certain user-specified interval of the current peak are considered when comparing the theoretical and experimental distributions.

AdductHunter uses Dynamic Time Warping (DTW) [37] to find the dissimilarity between distributions, that is, $\phi (\varvec{x},p)$ is the (Euclidean) distance between the optimally aligned theoretical and experimental isotopic distributions. DTW works by computing a distance matrix between the two isotopic distributions, where each cell in the matrix represents the distance between a specific point in one distribution and a specific point in the other distribution. The optimal path through the distance matrix that minimizes the total distance between the two distributions is then computed by constructing a cost matrix that accumulates the distances between all possible pairs of points in the two distributions. The cost matrix is then traversed in a way that minimizes the total accumulated cost along the path, that is, the optimally aligned dissimilarity between the two distributions.

### Output table

After the optimization problem is solved, a table of feasible protein adducts with an indication of the closest fit for each peak is returned (see Table 4). The table is sorted by experimental peak mass and closeness of fit (loss). Here, the theoretical peak mass is recorded as the most abundant isotopologue for a given species and is used to calculate mass error in ppm.

**Table 4:** Truncated output table for a spectrum recorded for a Ub/cisplatin containing feasible solutions and their corresponding measures

Identity	PO	Intensity	Mass	Peak	ppm	Closeness of Fit	Closest
Ub	0	0.823263	8564.634	8564.63	0.359771	0.331434	TRUE
Ub + Pt	2	0.025543	8757.589	8757.608	2.199949	0.602192	TRUE
Ub + Pt + NH3	2	0.204161	8774.61	8774.624	0.979775	0.569154	TRUE
Ub + Pt + H2O	2	0.204161	8775.6	8774.624	111.1512	1.737765	FALSE
$\vdots$	$\vdots$	$\vdots$	$\vdots$	$\vdots$	$\vdots$	$\vdots$	$\vdots$

The columns PO, Mass, and Peak, refer to the proton offset, theoretical nominal mass peak, and experimental mass peak, respectively

## Results and discussion

We examined the performance of AdductHunter on a variety of datasets to understand its effectiveness in accurately identifying protein adducts, as well as discuss here its limitations and further development.

AdductHunter was specifically developed to identify adducts formed between metal complexes and proteins. A collection of 22 unique datasets was analyzed to provide a comprehensive performance benchmark for AdductHunter (see Table 5). The metal complexes used were cisplatin, oxaliplatin, RAPTA-C, RM-175, Au-1, Au-2, and Au-3, with formulas cis-Pt($$\hbox {NH}_3$$)_2_$$\hbox {Cl}_2$$, Pt($$\hbox {C}_6$$$$\hbox {H}_{{14}}$$$$\hbox {N}_2$$)($$\hbox {C}_2$$$$\hbox {O}_4$$), Ru($$\eta ^6$$-$$\hbox {C}_{{10}}$$$$\hbox {H}_{{14}}$$)($$\hbox {PN}_3$$$$\hbox {C}_6$$$$\hbox {H}_{{12}}$$)$$\hbox {Cl}_2$$, [Ru($$\eta ^6$$-$$\hbox {C}_{{12}}$$$$\hbox {H}_{{10}}$$)($$\hbox {C}_{{2}}$$$$\hbox {H}_8$$$$\hbox {N}_2$$)Cl]$$\hbox {PF}_6$$, [Au($$\hbox {C}_{{19}}$$$$\hbox {H}_{{17}}$$$$\hbox {N}_2$$)(OH)]$$\hbox {PF}_6$$, Au($$\hbox {C}_{{12}}$$$$\hbox {H}_{{11}}$$$$\hbox {N}_2$$$$\hbox {O}_2$$)$$\hbox {Cl}_2$$, and Au($$\hbox {C}_{{12}}$$$$\hbox {H}_{{10}}$$N)$$\hbox {Cl}_2$$, respectively. The proteins used were cytochrome c (CyC, $$\hbox {C}_{{560}}$$$$\hbox {H}_{{874}}$$$$\hbox {Fe}_1$$$$\hbox {N}_{{148}}$$$$\hbox {O}_{{156}}$$$$\hbox {S}_4$$), ubiquitin (Ub, $$\hbox {C}_{{378}}$$$$\hbox {H}_{{629}}$$$$\hbox {N}_{{105}}$$$$\hbox {O}_{{118}}$$$$\hbox {S}_1$$), hen egg-white lysozyme (HEWL, $$\hbox {C}_{{613}}$$$$\hbox {H}_{{951}}$$$$\hbox {O}_{{185}}$$$$\hbox {N}_{{193}}$$$$\hbox {S}_{{10}}$$), and myoglobin (Mb, $$\hbox {C}_{{769}}$$$$\hbox {H}_{{1212}}$$$$\hbox {N}_{{210}}$$$$\hbox {O}_{{218}}$$$$\hbox {S}_2$$). Each data set contained a mixture of at least one protein and one metal complex (see Table 5). We compared the output from AdductHunter for each dataset against the corresponding ground truth, that is, the manually identified protein adducts.

**Table 5:** Information for all datasets used in this study. The metal complexes cisplatin, oxaliplatin, RAPTA-C, RM-175, Au-1, Au-2, and Au-3 have formulas cis-($$\hbox {NH}_3$$)_2_$$\hbox {PtCl}_2$$, Pt($$\hbox {C}_6$$$$\hbox {H}_{{14}}$$ $$\hbox {N}_2$$)($$\hbox {C}_2$$$$\hbox {O}_4$$), Ru($$\eta ^6$$-$$\hbox {C}_{{10}}$$$$\hbox {H}_{{14}}$$)($$\hbox {PN}_3$$$$\hbox {C}_6$$$$\hbox {H}_{{12}}$$)$$\hbox {Cl}_2$$, [Ru($$\eta ^6$$-$$\hbox {C}_{{12}}$$$$\hbox {H}_{{10}}$$)($$\hbox {C}_{{2}}$$$$\hbox {H}_8$$$$\hbox {N}_2$$)Cl]$$\hbox {PF}_6$$, [Au($$\hbox {C}_{{19}}$$$$\hbox {H}_{{17}}$$$$\hbox {N}_2$$)OH]$$\hbox {PF}_6$$, Au($$\hbox {C}_{{12}}$$$$\hbox {H}_{{11}}$$$$\hbox {N}_2$$$$\hbox {O}_2$$)$$\hbox {Cl}_2$$, and Au($$\hbox {C}_{{12}}$$$$\hbox {H}_{{10}}$$N)$$\hbox {Cl}_2$$, respectively

Metal complexes	Proteins	Instrument used	MIS	Reference
Cisplatin	CyC	FT-ICR	14	Unpublished data
	HEWL	FT-ICR	4	Unpublished data
	Mb	FT-ICR	19	Unpublished data
	Ub	FT-ICR	20	Unpublished data
	Ub	WA	20	[5]
	Mix	FT-ICR	13	Unpublished data
Oxaliplatin	CyC	FT-ICR	18	Unpublished data
	HEWL	FT-ICR	8	Unpublished data
	Mb	FT-ICR	11	Unpublished data
	Mb-H	FT-ICR	11	Unpublished data
	Ub	FT-ICR	5	Unpublished data
	Mix	FT-ICR	10	Unpublished data
RAPTA-C	CyC	FT-ICR	8	Unpublished data
	HEWL	FT-ICR	4	Unpublished data
	Mb	FT-ICR	19	Unpublished data
	Mb-H	FT-ICR	19	Unpublished data
	Ub	FT-ICR	12	Unpublished data
	Mix	FT-ICR	16	Unpublished data
RM-175	Ub	qTOF	2	[11]
Au-1	CyC, Ub	qTOF	10	[41]
Au-2	CyC, Ub	qTOF	23	[41]
Au-3	CyC, Ub	qTOF	32	[41]

The proteins cytochrome c (CyC), ubiquitin (Ub), hen egg-white lysozyme (HEWL), and myoglobin (Mb) have formulas $$\hbox {C}_{{560}}$$$$\hbox {H}_{{874}}$$$$\hbox {Fe}_1$$$$\hbox {N}_{{148}}$$$$\hbox {O}_{{156}}$$$$\hbox {S}_4$$, $$\hbox {C}_{{378}}$$$$\hbox {H}_{{629}}$$$$\hbox {N}_{{105}}$$$$\hbox {O}_{{118}}$$$$\hbox {S}_1$$, $$\hbox {C}_{{613}}$$$$\hbox {H}_{{951}}$$$$\hbox {O}_{{185}}$$$$\hbox {N}_{{193}}$$$$\hbox {S}_{{10}}$$, and $$\hbox {C}_{{769}}$$$$\hbox {H}_{{1212}}$$$$\hbox {N}_{{210}}$$$$\hbox {O}_{{218}}$$$$\hbox {S}_2$$, respectively

The instruments used were Bruker Solarix 7T FT-ICR (FT-ICR), Waters QToF Ultima API (WA) and Bruker maXis qTOF (qTOF) mass spectrometers, with deconvolution resolutions of 100,000, 25,000, and 30,000, respectively

Under the Proteins column, Mix refers to an equimolar mix of all proteins; HEWL, CyC, Ub, and Mb

Mb-H refers to the same dataset directly above, but with a higher sampling rate

MIS refers to manually identified species, that is, the number of ground truth species

### Peak identification

**Fig. 4:**
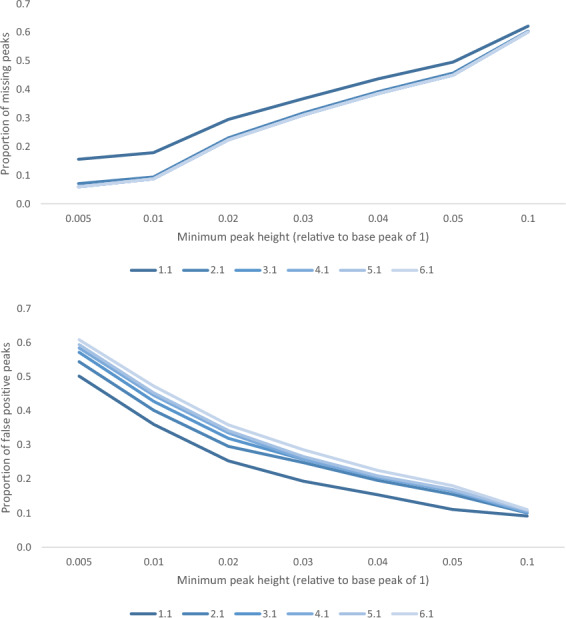
Missing (top) and false positive (bottom) proportion of peaks at different minimum peak heights for increasing tolerance values

Peak detection in mass spectra is subject to identifying many false positives, especially at low intensities where noise is prevalent. Here, we define false positives as peaks detected by the tool but not ground truth peaks, and false negatives as ground truth peaks that were not picked up by the tool. The peak detection algorithm in AdductHunter is highly sensitive to the normalized minimum peak height. A lower minimum peak height allows AdductHunter to detect more manually identified peaks, although with diminishing returns and increasing false positives (see Fig. 4). Through testing and assuming an equal weight on false positives and false negatives, a value of 0.01 was found to be optimal; decreasing the setting to 0.005 added many false positives with few manually identified peaks, likely due to noise, and increasing the value to 0.02 removed a notable portion of manually identified peaks with a less significant reduction in false positives. Another notable parameter in peak detection is the minimum distance between two (manually identified) adjacent peaks, found to be 15.9 Da over all datasets and set to 15 as a default.

The other significant parameter to be defined is the tolerance around peaks, *t*. Peak tolerance makes strides at accounting for noise in mass spectra and error in the mass approximation of the adduct in AdductHunter. Here, individual compound masses are summed instead of finding the most abundant isotopologue for the adduct, which is a non-linear, non-continuous, and computationally-expensive calculation. Consequently, we decided to keep the formulation linear as it is a close approximation of the true mass value. This parameter has the most flexibility, uncertainty, and, alongside the minimum peak height, is where computational efficiency in the constraint optimization is most affected.

**Fig. 5:**
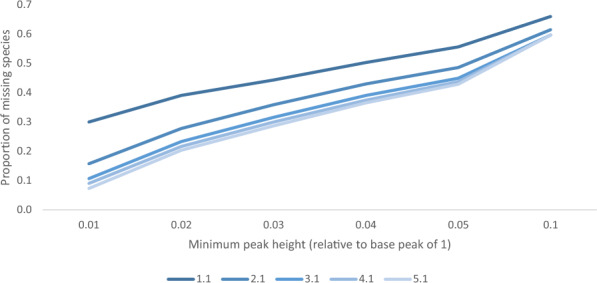
Missing proportion of species which improves as the tolerance is increased, albeit with diminishing returns in accuracy

Since *t* is directly proportional to the size of the feasible set, a large enough *t* is desired to be confident that as many manually identified species are captured in the feasible set, but not so large that numerous unwanted species are made feasible (see Fig. 5). Recall that only peaks with at least one feasible solution are returned in the output (and the best-fit species is found for each peak). As a result, a larger *t* will not only return more potential species, but more unique peaks as well; this brings about the detection of peaks as false positives that would not have been returned with a lower tolerance. Tolerance values were selected to be slightly larger than multiples of the atomic mass of a hydrogen atom at 1.008, that is, *n*H where $n \in \mathbb {N}$, which has been approximated to $n + 0.1$. It was found that for the given data and tolerances greater than 3.1, no more peaks in the manually identified were returned, meaning the missing number of manually identified peaks did not change. Hence, increasing the tolerance past this point means new peaks returned are all false positives.

### Default parameters

The results of the benchmarking tests were used to set the default parameters values for AdductHunter. As a broader range of data is tested and analyzed, a parameter search would prove useful to precisely determine their optimal values. Parameter values could also be made variable and dependent on the mass. The assignment of proton adducts became more challenging for higher adducts with larger masses, as they tended to be further away from the experimental peak, making reliable identification difficult. In the used datasets, higher mass adducts at lower intensity in the mass spectra and the peaks usually were surrounded by increased noise and complexity, which comes naturally with more individual components involved in each adduct. Thus, for peaks at higher masses, parameters may need to accommodate for an increased feasible set to capture the previously identified peaks in the ground truth. For example, the peak tolerance could increase as the mass of the protein adducts increases, with possibly a smaller starting peak tolerance than the constant mass tolerance (3.1) used as mass error increases with adduction complexity. Future work may also include automatically calculating the noise threshold as a function of the baseline intensity and noise level of the spectrum, instead of being a user-defined input.

### Objective function analysis

A variety of established similarity measures for the objective function were tested over all datasets to determine which metric would work best. We used the “similaritymeasures” package [38] to test the following measures: the area between curves [38], Partial Curve Matching [39], discrete Fréchet distance [40], and Dynamic Time Warping [37] with Euclidean and City Block (Manhattan) distance measures. The normalized intensity values were also scaled by a range of weights – 0 (effectively only using mass), $10^{-1}$, $10^0$, $10^1$, $10^2$, $10^3$ – to understand its significance in finding the best fit. Dynamic Time Warping with an Euclidean distance measure was found to have the best average performance with a weight of $10^{-1}$ on the intensity. However, other (tested) similarity metrics and weights may be used depending on the data (see Table 6 and Additional File 3). As noise affects the experimental intensities, more weight is applied to mass accuracy when comparing experimental and theoretical distributions. Using only mass however, performs poorly due to multiple feasible solutions having similar mass values.

**Table 6:** Mean accuracy across all datasets using the hyperfine isotope generator for tested metrics at different weights on the intensity

Metric	Weight (on intensity)					
	0	0.1	1	10	100	1000
Area between Curves	0.600	*0.713*	0.707	0.697	0.697	0.697
Discrete Fréchet Distance	0.600	0.701	*0.740*	0.725	0.722	0.722
Partial Curve Matching	0.600	*0.699*	–	–	–	–
Dynamic Time Warping-E	0.600	***0.842***	0.772	0.739	0.737	0.737
Dynamic Time Warping-CB	0.600	*0.837*	0.775	*0.747*	0.739	0.740

The Partial Curve Matching measure normalizes both mass and intensity inputs to the same scale, meaning different non-zero weights on the intensity have the same effect. Dynamic Time Warping-E and Dynamic Time Warping-CB refer to the Dynamic Time Warping with Euclidean and City Block (Manhattan) distance measures, respectively

The best value for each metric is in italics and the overall best is in bold

A different type of objective function initially considered was to measure the mass error (ppm) at each peak *p*, which is a scaled form of the relative error between the peaks in the theoretical isotope pattern and experimental isotope distribution:

12 $$\begin{aligned} ppm(\varvec{x}, p) = \bigg | \frac{T(\varvec{x}) - p}{T(\varvec{x})} \bigg | \times 10^6, \end{aligned}$$

where $T(\varvec{x})$ is the theoretical peak mass of species $\varvec{x}$.

Parts per million error calculations are a common approach in MS analyses [4], and would be substantially easier to implement and interpret than a distance metric. However, the linear mass approximation used to find theoretical mass peaks means that ppm would need to be measured after finding the feasible set to accurately calculate its value (as the isotope pattern is needed to find its peak, which is a non-linear process), hence it is unusable as an objective in the constraint formulation. Furthermore, using a full isotope pattern is more robust as there are cases where two consecutive isotope peaks have near identical abundance in the experimental spectrum, and so measured and theoretical distributions may disagree on the identity of the tallest peak, resulting in large ppm values.

### Running time

Experiments were run using an Intel Core i5-8250U CPU and 8GB RAM. When calculating the objective, the total time taken for the AdductHunter analysis of a recorded spectrum was dominated by the generation of hyperfine isotopic mass distributions. In contrast, the choice of objective function had a negligible effect on the total analysis time. Across all datasets, generating the hyperfine isotope distribution took approximately 135.5 s on average. The time required to identify peaks and generate the set of feasible species pales in comparison, taking approximately 0.41 s on average. Additionally, an approximate, coarse method for generating isotopic mass distributions exists in pyOpenMS that is significantly faster ($\sim$85 times) than generating the hyperfine peaks, which took approximately 1.68 s on average. However, the mass values calculated using the coarse method will not accurately reflect the most abundant isotopologue peak as a simplified formula is used to find isotope peaks with greater mass [17]. This imprecision leads to decreased accuracy and high error values for almost all metrics at an intensity weight of 10^-1^, although some improvements can be seen for larger intensity weights across all metrics (see Table 7). The best performance (mean accuracy of 0.787) was achieved with the Dynamic Time Warping with City Block (Manhattan) distance measures at an intensity weight of 10^0^. However, it is worse than than the one achieved with the hyperfine method (mean accuracy of 0.842, see Table 6). Hence, we recommend to use the hyperfine method, although the coarse method may be used for rapid preliminary testing. As species and peaks generated are independent of each other, further improvement on the analysis time would involve parallelizing the generation of isotope patterns, constraint integer optimization formulations, and objective function calculations.

**Table 7:** Mean accuracy across all datasets using the coarse isotope generator for tested metrics at different weights on the intensity

Metric	Weight (on intensity)					
	0	0.1	1	10	100	1000
Area between Curves	0.600	*0.708*	0.697	0.691	0.691	0.691
Discrete Fréchet Distance	0.600	0.639	0.719	*0.743*	0.740	0.740
Partial Curve Matching	0.600	*0.749*	–	–	–	–
Dynamic Time Warping-E	0.600	0.777	0.774	0.778	*0.781*	0.775
Dynamic Time Warping-CB	0.600	0.779	0.782	***0.787***	0.777	0.778

The best value for each metric is in italics and the overall best is in bold

The Partial Curve Matching measure normalizes both mass and intensity inputs to the same scale, meaning different non-zero weights on the intensity have the same effect. Dynamic Time Warping-E and Dynamic Time Warping-CB refer to the Dynamic Time Warping with Euclidean and City Block (Manhattan) distance measures, respectively

## Conclusion

AdductHunter was created to identify protein-metal complex adducts in deconvoluted mass spectrometry data by formulating a constraint integer optimization problem at each experimental mass peak and using dynamic time warping to find the best fit species based on its theoretical isotopic distribution. The results presented herein provide comprehensive evidence that AdductHunter effectively detects peaks within mass spectrometry data and accurately determines their speciation much faster than interpreting the spectra manually. Efforts are currently underway to address AdductHunter’s limitations, specifically by introducing the deconvolution of experimental mass spectra as well as ensuring that it can appropriately handle samples with more than one metal complex in the incubation mixture.

### Supplementary Information

**Additional file 1.** Species description and constraints input CSV file for cytochrome c incubated with cisplatin.

**Additional file 2.** Standard adducts’ descriptions and constraints input CSV file.

**Additional file 3.** Accuracies for each dataset using the hyperfine isotope generator with different similarity measures and weights. Metrics from left to right: area between curves, Partial Curve Matching, discrete Fréchet distance, and Dynamic Time Warping with Euclidean and City Block (Manhattan) distance measures, respectively. Datasets are grouped by metal complexes and proteins (see Table 5 for more detail).

## Data Availability

AdductHunter is freely accessible on Github under an open-source (MIT) license at github.com/dlon450/MS-Protein-Adduct-Identification, and can also be found at adducthunter.wickerlab.org. Scripts used for the results section can be found at github.com/dlon450/MS-Protein-Adduct-Identification/tree/main/src. Finally, not all data sets are available as some are currently unpublished (see Table 5 for more information).

## Footnotes

1 www.nesvilab.org/software.html.

2 www.github.com/dlon450/MS-Protein-Adduct-Identification.
